# Supplementary material for: Health benefits attributed to 17α-estradiol, a lifespan-extending compound, are mediated through estrogen receptor α
Source: eLife. 2020 Dec 8;9:e59616. doi: 10.7554/eLife.59616 (PMC7744101; doi:10.7554/eLife.59616)
Supplement: Transparent reporting form [file elife-59616-transrepform.docx]

***eLife’s* transparent reporting form**

We encourage authors to provide detailed information *within their submission* to facilitate the interpretation and replication of experiments. Authors can upload supporting documentation to indicate the use of appropriate reporting guidelines for health-related research (see [EQUATOR Network](http://www.equator-network.org/%20)), life science research (see the [BioSharing Information Resource](https://biosharing.org/)), or the [ARRIVE guidelines](http://www.plosbiology.org/article/info:doi/10.1371/journal.pbio.1000412) for reporting work involving animal research. Where applicable, authors should refer to any relevant reporting standards documents in this form.

If you have any questions, please consult our Journal Policies and/or contact us: [editorial@elifesciences.org](mailto:editorial@elifesciences.org).

**Sample-size estimation**

- You should state whether an appropriate sample size was computed when the study was being designed
- You should state the statistical method of sample size computation and any required assumptions
- If no explicit power analysis was used, you should describe how you decided what sample (replicate) size (number) to use

Please outline where this information can be found within the submission (e.g., sections or figure legends), or explain why this information doesn’t apply to your submission:

The ChIP-Seq and RNA-Seq experiments were designed based on field best practices and ENCODE guidelines. ChIP peak comparisons use a false discovery rate correction as does the RNA-Seq study for differential gene expression calling. Sample size calculations for these types of studies are very difficult to perform accurately and n=3 was performed per group based on a prior experience that these are sufficient to detect differences.

The ERαKO experiments were designed as 2x2 analyses (two-Way ANOVA) within sex. In male ERαKO experiments, a WT Chow group was also evaluated for visual comparison purposes but was not included in statistical comparisons. We determined that our minimal sample size should be n=7/group based on the variances in adiposity changes and fasting insulin from previous studies. We predicted we would detect a difference of 20% at a power of 0.8 with an alpha of 0.05.

The hyperinsulinemic-euglycemic clamp experiments were designed as single 2-group comparison (Student’s t-test) studies. We determined that our minimal sample size should be n=6/group based on the variances in glucose infusion rates from previous studies. We predicted we would detect a difference of 20% at a power of 0.8 with an alpha of 0.05.

**Replicates**

- You should report how often each experiment was performed
- You should include a definition of biological versus technical replication
- The data obtained should be provided and sufficient information should be provided to indicate the number of independent biological and/or technical replicates
- If you encountered any outliers, you should describe how these were handled
- Criteria for exclusion/inclusion of data should be clearly stated
- High-throughput sequence data should be uploaded before submission, with a private link for reviewers provided (these are available from both GEO and ArrayExpress)

Please outline where this information can be found within the submission (e.g., sections or figure legends), or explain why this information doesn’t apply to your submission:

The timeline and frequency of experiments are listed in the figure legends.

Our reported n/group is an indicator of biological replicates and our data is presented as mean ± SEM. Each mouse within each study is considered a biological replicate. To provide the reader evidence of biological replicates and variance within the group, we also display individual biological replicates over the bar graphs throughout the manuscript.

For glucose measures, each biological replicate was generated from an average of 2-4 technical replicates, which were taken within seconds of each other. If the first two technical replicates displayed differences greater than 20mg/dL, additional samples were taken until at least 2 technical replicates were within 20mg/dL of one another and the outlying values were excluded from calculations. For fasting insulin and qPCR data collection, each biological replicate was a product of the average of 2 technical replicates. Oil Red O quantification utilized 10 technical replicate images within each biological replicate.

Individual data points are shown on the figures and sample sizes are listed in the figure legends. No data exclusions were performed except for:

1. During glucose tolerance testing, a few mice were noted during the blinded procedure as being visibly stressed, which is known to acutely affect their blood glucose levels. As such, mice 12, 43, 22, 23, and 27 were excluded from the analysis.

2. Liver cDNA from mouse IDs 10, 15, 25, and 40 livers failed to demonstrate qPCR amplification in housekeeping and target genes so they were excluded from the analyses.

3. Rats (IDs C2, C4, C7, E7) undergoing hyperinsulinemic-euglycemic clamp experiments in conjunction with ICV infusions that displayed hepatic glucose production rates (*R*_a_) less than 0.05 mg/kg-min were excluded from the analyses. Rates this low are suggestive of a problem with the animal due to this being anywhere from 6-10 times lower than hepatic glucose production rates in all other rats being analyzed. These 4 rats were excluded entirely from all data analyses based on suspicion of issues related to surgical stress.

The full complement of ChIP-Seq and RNA-Seq data is publicly available at: <https://www.ncbi.nlm.nih.gov/geo/query/acc.cgi?acc=GSE151039>

**Statistical reporting**

- Statistical analysis methods should be described and justified
- Raw data should be presented in figures whenever informative to do so (typically when N per group is less than 10)
- For each experiment, you should identify the statistical tests used, exact values of N, definitions of center, methods of multiple test correction, and dispersion and precision measures (e.g., mean, median, SD, SEM, confidence intervals; and, for the major substantive results, a measure of effect size (e.g., Pearson's r, Cohen's d)
- Report exact p-values wherever possible alongside the summary statistics and 95% confidence intervals. These should be reported for all key questions and not only when the p-value is less than 0.05.

Please outline where this information can be found within the submission (e.g., sections or figure legends), or explain why this information doesn’t apply to your submission:

Statistical analyses are detailed at the end of the methods section as well as within the figure legends. Results are presented as mean ± SEM unless otherwise stated with p values less than 0.05 considered to be significant unless otherwise specified. Analyses of differences between groups were performed by 2-way ANOVA, 2-way repeated measures ANOVA, or Student’s t-test where appropriate using SigmaPlot 12.5 Software. A Benjamini-Hochberg multiple testing correction was applied to the F test result to correct for the number of transcripts, proteins, and fatty acids analyzed.

ChIP-Seq analyses are detailed in the methods, results, and figure legends. Reads were aligned to the human genome (hg19) using bowtie2 and duplicated reads were flagged with Picard-tools. ERα binding peaks were called using MACS2 with recommended settings. Peak genomic location, breadth of coverage, and peak summit location were determined using MACS2. NarrowPeak files containing peak information were used to determine differential ERα binding. First, peaks were centralized around the summit and 250bp flanking regions were added to the summit location to generate equal 500bp regions across all experimental groups. Peak files were then used to extract read counts from the aligned de-duplicated BAM file using samtools, read counts were then normalized to total library sequencing depth. To determine differential binding, the R package diffbind was utilized. First, normalized read counts were log2 transformed and normalized across all experimental groups. Differential binding between treatment groups was determined using negative binomial regression models utilized in the R package DESeq2, statistical significance for pairwise comparisons between experimental groups was determined using Wald test. To account for multiple comparisons, we used Benjamini-Hochberg multiple testing correction (False-discovery rate, FDR). Significance threshold was set to false-discovery rate (FDR) corrected p-value < 0.05. Motif analysis was performed using HOMER with standard settings. Peak regions called for each treatment group were analyzed to identify enriched motifs relative to the entire genome. Hypergeometric test was used to test enrichment. Only motifs with FDR corrected p-value < 0.05 were reported as significant. For pairwise differential motif enrichment or depletion across experimental group we utilized the hypergeometric test by using the number of sequences with motif from each group and total number of peaks as total sample size. Motifs that appear in less than 5 sequences between both test groups were removed. Benjamini-Hochberg multiple testing correction was utilized to control for false discovery rate (FDR < 0.05).

RNA-Seq analyses are detailed in the methods, results, and figure legends. Sequence quality control was performed with fastQC, Paired reads were trimmed using trimmomatic, and were aligned to the hg19 genome using STAR. Differential expression was determined using previously described methods. In brief, gene counts were determined with the R package GenomicAlignments ‘summarizeOverlap’ function. Gene counts were then transformed using regularized log transformation and normalized relative to library size using the DESeq2 R package. Differential expression was determined using negative binomial generalized linear model using *counts ~ treatment* model. We performed pairwise differential expression between all experimental groups using Wald test. All comparisons were corrected for multiple testing using Benjamini-Hochberg multiple testing correction method. Differential expression significance threshold was set to FDR corrected p-value < 0.05.

n/group are displayed within the figures legends as well as biological replicate values overlaid on the bar graphs in all figures.

(For large datasets, or papers with a very large number of statistical tests, you may upload a single table file with tests, Ns, etc., with reference to sections in the manuscript.)

**Group allocation**

- Indicate how samples were allocated into experimental groups (in the case of clinical studies, please specify allocation to treatment method); if randomization was used, please also state if restricted randomization was applied
- Indicate if masking was used during group allocation, data collection and/or data analysis

Please outline where this information can be found within the submission (e.g., sections or figure legends), or explain why this information doesn’t apply to your submission:

Mice for the ERαKO and WT Littermate experiments were randomized into treatment groups (HFD or HFD+17α) by mass, food intake, body composition, fasting glucose, and fasting insulin prior to beginning treatment so that the average of each treatment group started with near identical values.

Rats for the hyperinsulinemic-euyglycemic clamp experiments were randomly assigned into control or treatment groups.

When possible, blinding was practiced so that samples and analyses were processed/performed without bias and at some stage by different authors, who only received a code (mouse ID number) for sample identification. For instance: A.R.R., R.S., and S.A-M. were blinded to the identity of the mice during *in vivo* metabolic data collection. S.N.M. and A.R.R. were blinded to the identity of histological samples used for Oil Red O imaging and quantification. M-P.A. was blinded to the identity of liver tissues for fatty acid analyses (performed by M-P.A.). Eicosanoid analyses by UCSD were performed under blinded conditions. Blinding was also applied at the stage of ChIP-Seq and RNA-Seq analyses. De-identified groups were unmasked after statistical analyses were performed.

**Additional data files (“source data”)**

- We encourage you to upload relevant additional data files, such as numerical data that are represented as a graph in a figure, or as a summary table
- Where provided, these should be in the most useful format, and they can be uploaded as “Source data” files linked to a main figure or table
- Include model definition files including the full list of parameters used
- Include code used for data analysis (e.g., R, MatLab)
- Avoid stating that data files are “available upon request”

Please indicate the figures or tables for which source data files have been provided:

The full complement of ChIP-Seq and RNA-Seq data is publicly available at: <https://www.ncbi.nlm.nih.gov/geo/query/acc.cgi?acc=GSE151039>

We have also provided an additional data as Supplementary Files 1 and 2 that includes quantitation of ERα binding peaks and all identified ERα DNA binding motifs following exposure to 17α-E2, 17β-E2, and Vehicle treatment, respectively. Supplementary File 3 contains the full complement of circulating eicosanoid data.
